# Exploring the mechanisms of protective effect of high-energy X-ray FLASH radiotherapy on intestine through multi omics analysis

**DOI:** 10.1186/s13014-025-02763-z

**Published:** 2025-11-27

**Authors:** Huan Du, Binwei Lin, Yihan Zhu, Xiaofei Hao, Mingming Tang, Wei Wu, Decai Wang, Yiwei Yang, Yuwen Liang, Wenqiang Tang, Haonan Xu, Jie Li, Feng Gao, Xiaobo Du

**Affiliations:** 1 School of Medicine, Mianyang Central Hospital, University of Electronic Science and Technology of China, Mianyang, 621000 China; 2 NHC Key Laboratory of Nuclear Technology Medical Transformation, School of Medicine, Mianyang Central Hospital, University of Electronic Science and Technology of China, Mianyang, 621000 China; 3 Sichuan Clinical Research Center for Radiation and Therapy, Mianyang Central Hospital, Mianyang, 621000 China; 4 Institute of Applied Electronics, China Academy of Engineering Physics, Mianyang, 621900 China; 5 Clinical Medical School, North Sichuan Medical College, Nanchong, 637000 P.R. China; 6 Southwest University of Science and Technology, Mianyang, 621010 P.R. China

**Keywords:** X-ray FLASH, FLASH effect, Intestine, Metagenomics, Untargeted metabolic, Single-cell seq

## Abstract

**Background:**

The aim of this study is to investigate the potential mechanisms underlying the protective effects of high-energy X-ray FLASH radiotherapy (FLASH-RT) on intestine through multi-omics analysis.

**Methods:**

This study utilized syngeneic colon carcinoma mouse models of CT26 and MC38 to evaluate the therapeutic efficacy of FLASH-RT versus conventional dose rate radiotherapy (CONV-RT) by monitoring survival, tumor size, and body weight. Furthermore, healthy C57BL/6 female mice received whole-abdominal irradiation with either FLASH-RT, CONV-RT, or sham irradiation to compare differences in normal tissue protection. 72 h post-irradiation, intestinal contents from mice were collected for metagenomic analysis, and intestinal tissue was harvested for non-targeted metabolic and single-cell sequencing analyses.

**Results:**

In CT26 and MC38 models, both CONV-RT and FLASH-RT have demonstrated similar anti-tumor efficacy. Compared with CONV-RT, whole-abdominal FLASH-RT significantly alleviated acute intestinal injury in mice, as evidenced by better preservation of crypt numbers and villus architecture in the FLASH group. Metagenomic analysis revealed that the relative abundance of the gut-protective bacterium *Ligilactobacillus ruminis* was significantly higher in the FLASH group than in the CONVgroup. Non-targeted metabolomic profiling identified 34 differential metabolites, of which 29 were upregulated and 5 were downregulated in the FLASH group. Notably, the abundance of 2-hydroxyglutarate, a metabolite associated with the butyrate metabolism pathway, was significantly elevated in the FLASH group compared with the CONV group (*p* < 0.05). Single-cell sequencing data revealed notable differences in cell distribution and proportions between the groups, with a higher proportion of fibroblasts, proliferative cells, macrophages, and CD4 + T cells in the FLASH group compared to the CONV and control groups. Immunofluorescence analysis revealed a significantly greater number of Lgr5⁺ intestinal stem cells in the FLASH group compared to the CONV group. Conversely, immunohistochemical analysis demonstrated stronger p50/p65 staining intensity in the CONV group relative to the FLASH group.

**Conclusions:**

This study confirms that FLASH-RT, compared to CONV-RT, maintains equivalent antitumor efficacy while mitigating damage to normal intestinal tissues. Moreover, it preliminarily reveals that the protective mechanism of FLASH-RT is multifaceted, involving remodeling of the microbiota-metabolite axis, attenuation of inflammatory responses, and enhanced preservation of stem cells.

**Supplementary Information:**

The online version contains supplementary material available at 10.1186/s13014-025-02763-z.

## Introduction

Radiotherapy is a key component of comprehensive cancer treatment [1], with approximately 60%-70% of cancer patients requiring radiotherapy at various stages of their disease [2, 3]. However, for patients with abdominal and pelvic tumors, the incidence of acute radiation enteritis is as high as 60%-80%, making it one of the primary factors limiting the application of radiotherapy [4, 5]. Currently, there are no effective preventive or therapeutic measures [6]. Therefore, it is crucial to explore radiotherapy methods that can alleviate acute radiation enteritis and elucidate their underlying mechanisms. This research is essential for advancing the broader use of radiotherapy in the treatment of abdominal and pelvic tumors.

FLASH radiotherapy (FLASH-RT) is an ultra-high dose rate radiation therapy technique that synergistically optimizes physical parameters [7], including instantaneous dose rate (typically >10^6^ Gy/s) [8], mean dose rate (conventionally >40 Gy/s) [9], dose per pulse (>4 Gy) [7], total beam-on time (< 100 ms) [10], and other parameters, to achieve significant normal tissue protection while maintaining antitumor efficacy in vivo [9]. Compared to conventional dose-rate radiotherapy (CONV-RT), FLASH-RT offers two significant advantages: first, it drastically shortens treatment time, reducing what would typically take several weeks of treatment to a millisecond scale; second, it provides better protection for normal tissues while maintaining effective tumor cell killing [11]. Several hypotheses regarding the mechanisms of the FLASH effect have been proposed, including oxygen depletion, oxidative stress, DNA damage repair, and immune responses [12–16]. However, these factors are interrelated, and the precise mechanism of the FLASH effect remains unclear, requiring further research for exploration.

Electrons, low-energy X-rays, and protons have been used in preclinical studies of FLASH-RT. However, these types of radiation have not been widely used in clinical practice due to limitations in energy and cost [17–21]. High-energy X-rays, characterized by their deep penetration, narrow divergence, low radiation intensity, and relatively low cost, are the most commonly used form of radiation in clinical radiotherapy [22]. Nevertheless, the inherent difficulty in generating high-energy, ultra-high-dose-rate X-rays has hindered progress in this research area [23]. This study utilizes a compact single high-energy X-ray source (CHEXs) FLASH-RT device for FLASH-RT to confirm FLASH effects. Additionally, through multi-omics analysis, the study explores the potential mechanisms underlying the protective effect of high-energy X-ray FLASH-RT on intestine.

## Materials and methods

### Irradiation device and dosimetry

**Fig. 1:**
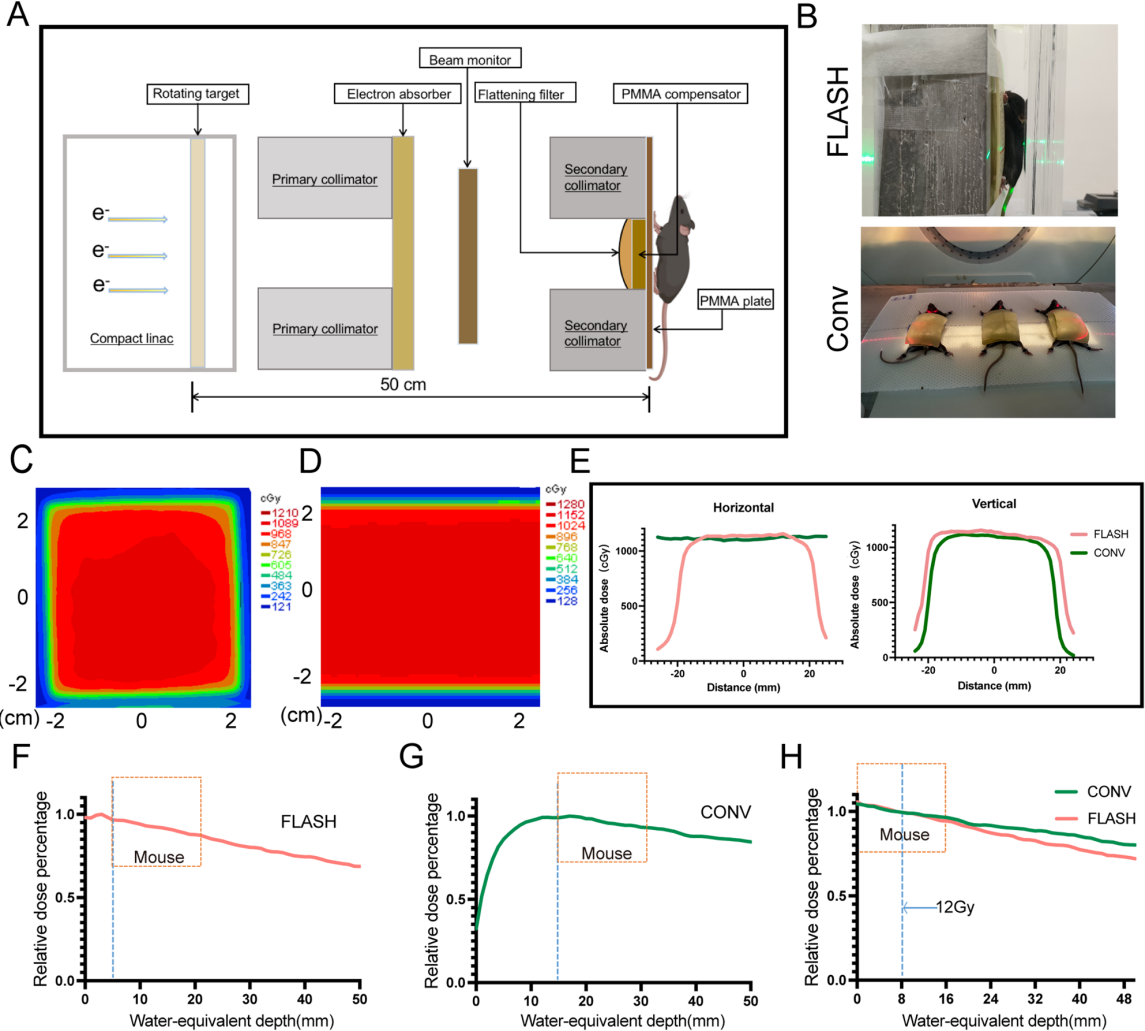
Parameters and dosimetry of FLASH-RT and CONV-RT. (**A**) Schematic of in vivo FLASH-RT experiment. (**B**) FLASH-RT and CONV-RT setup diagrams for total abdominal irradiation. **C**-**D**. Dose distribution for FLASH-RT and CONV-RT with a 4 × 4 field and a dose of 12 Gy. **E**.The horizontal and vertical dose profiles, representing the dose distribution in FLASH-RT and CONV-RT. **F**. Percentage Depth Dose (PDD) curve for X-ray source in FLASH-RT. **G**. PDD curve for X-ray source in Convr-RT. H. A dose of 12 Gy was delivered at 0.8 cm below the surface, showing comparable PDD curves between the FLASH-RT and CONV-RT

The CHEXs device (Zhongjiu Flash Medical Technology Co., Ltd.) was used for the FLASH-RT experiments (Fig. 1A). A clinically used 6 MV Elekta Precision linear accelerator (Elekta AB, Stockholm, Sweden) was employed for the CONV-RT experiments. A current transformer (BCT) was used to monitor beam current, and a diamond detector was installed downstream of the primary collimator for X-ray beam monitoring. Additionally, Gafchromic™ EBT-XD radiographic film (Ashland Inc., Covington, Kentucky, USA) was placed at the center of the irradiated target area, submerged in solid water, to monitor the dose distribution. The dose monitoring procedure followed previously established protocols [23]. The treatment setup for FLASH and CONV whole-abdomen irradiation is shown in Fig. 1B. The dose distribution for 12 Gy and the PDD curves are presented in Fig. 1C-H.

### Mice and tumor-bearing mouse model

Female C57BL/6J (RRID: MGI:3028467) and BALB/c (RRID: MGI:2161072) mice, aged 6–8 weeks, were obtained from Sibeifu Experimental Animal Technology Co., Ltd. (Beijing, China). Colon cancer cell lines CT26 and MC38 were sourced from ATCC (Manassas, Virginia, USA). Cells were cultured under standard conditions (37 °C, 5% CO_2_) until sufficient quantities were reached for tumor inoculation. For MC38 cells, 5 × 10⁵ cells were injected into the right posterior flank of C57BL/6J mice. For CT26 cells, 5 × 10^5^ cells were inoculated into the right posterior flank of BALB/c mice. Tumors were irradiated when the tumor volume reached 200 mm³. All animal experiments were conducted in accordance with the relevant ethical guidelines and were approved by the Animal Ethics Committee of Mianyang Central Hospital (approval number: S20230204).

### Tumor irradiation

Subcutaneous tumor models MC38 and CT26 were randomly divided into three groups: Control group (MC38: 0 Gy; CT26: 0 Gy), FLASH group (MC38: 15 Gy; CT26: 16.5 Gy), and CONV group (MC38: 15 Gy; CT26: 16.5 Gy) [24, 25]. A field size of 1.2 cm (craniocaudal) × 2 cm (lateral) was used to irradiate the tumors. The dose rates for FLASH-RT and CONV-RT were 244 Gy/s and 0.07 Gy/s, respectively. Mice were anesthetized with isoflurane and fixed on a solid water plate using adhesive tape. The radiation field was centered on the tumor site for irradiation. Mouse body weight and tumor volume were monitored twice weekly by the same researcher, and tumor volume was calculated using the formula: (length×width^2^)/2 [26]. Mice were euthanized when the tumor volume reached ≥ 2000 mm³ or if weight loss exceeded 20%.

### Whole abdominal irradiation of normal mice

For whole abdomen irradiation, the C57BL/6J mice were randomly divided into three groups: control (0 Gy), FLASH (12 Gy), and CONV (12 Gy). The irradiation field, measuring 4 cm (cranial) × 4 cm (lateral), was positioned with its upper boundary at the lower margin of the lungs (2 cm below the ear edges). The dose rates for FLASH-RT and CONV-RT were 334 Gy/s and 0.07 Gy/s, respectively. 72 h post-irradiation, five mice from each group were euthanized for tissue collection and subsequent analysis.

### HE staining

72 h post-irradiation, mice subjected to whole abdomen irradiation were euthanized, and intestinal tissue was harvested and washed with saline. The intestinal segments were dissected using fine scissors, rolled from the posterior end with the lumen facing outward [27]. The samples were fixed in formalin overnight, embedded in paraffin, and sectioned at 5 μm for H&E staining. A modified Swiss-roll crypt assay was employed to quantitatively assess acute crypt damage induced by ionizing radiation [28]. The most severely damaged areas were identified based on two independent assessments. The total number of crypts in regions larger than 3 mm was calculated, and only those with more than 10 cells and no signs of apoptosis were considered regenerative. Chiu’s score was used to assess the degree of intestinal tissue injury, classifying it into five levels (0–5). Level 0 is defined as normal mucosa, while Level 1 is characterized by the formation of subepithelial spaces at the tips of the villi. At Level 2, the subepithelial space is further expanded. At Level 3, extensive detachment of epithelial cells occurs on both sides of the villi. At Level 4, villus epithelium is shed. Finally, at Level 5, complete necrosis of the intestinal mucosa is observed, accompanied by ulceration and bleeding [29].

### Immunohistochemical

Fixed intestinal tissue samples were initially dehydrated using a series of ethanol solutions at graded concentrations (70%, 80%, 95%, and 100%), with each concentration incubated for 10 min. Following dehydration, the tissue samples were embedded in paraffin according to standard procedures. The embedded tissue blocks were then sectioned into thin slices (4–6 μm) using a rotary microtome. Paraffin-embedded tissue sections were deparaffinized sequentially in three steps using environmentally friendly deparaffinization reagents, each for 10 min. The sections were then rehydrated through a graded ethanol series of absolute ethanol, 95% ethanol, and 75% ethanol, each for 5 min, followed by three washes in distilled water, each for 3 min. Antigen retrieval was performed using high-pressure heat-induced epitope retrieval with EDTA buffer (pH 9.0) in a pressure cooker for 1 min and 30 s, followed by natural cooling and three washes with TBST. Endogenous peroxidase activity was blocked by incubating the sections in 3% hydrogen peroxide at room temperature in the dark for 30 min, followed by rinsing with distilled water. After outlining the tissue with a hydrophobic barrier pen, the sections were blocked with 10% serum at room temperature for 30 min. The serum was removed, and the primary antibody was diluted in 10% serum and applied at a volume of 50–100 µL per section, depending on tissue size, and incubated overnight at 4 °C. On the following day, sections were equilibrated to room temperature for 15 min, washed three times with TBST, and then incubated with the secondary antibody (diluted in TBST, 50–100 µL per section) at 37 °C for 45 min, followed by another round of washing. DAB substrate solution (freshly prepared, 50–100 µL per section) was added for color development, monitored under a microscope, and the reaction was stopped with tap water upon optimal staining. Nuclei were counterstained with hematoxylin for 1 min, differentiated with acid alcohol for 1–2 s, and blued for several seconds, followed by rinsing. The stained slides were dehydrated in two changes of absolute ethanol (2 min each), air-dried or oven-dried at 37 °C, and cover slipped using an environmentally friendly mounting medium or neutral resin. Microscopic examination and image acquisition were performed using an Olympus CX-31 upright microscope.(Ki67 Polyclonal Antibody: RRID: AB_2884867, 1:200 dilution); Cleave-caspase3: RRID: AB_2910623,1:500 dilution; a-SMA: RRID: AB_2811044; CD19: RRID: AB_2864513, 1:500 dilution; p50 Polyclonal Antibody: RRID: AB_2833407, 1:200 dilution; p65 Polyclonal Antibody: RRID: AB_87620, 1:200 dilution.

### Immunofluorescence

The paraffin-embedded tissue sections were deparaffinized using a series of environmental xylene agents for 10 min each, followed by hydration with ethanol (100%, 95%, 75%) for 5 min each, and washed three times with distilled water for 3 min. After antigen retrieval, tissue sections were subjected to high-pressure heat treatment using EDTA (pH 9.0) antigen retrieval solution, with the solution covering the tissue. The tissue was heated under pressure in the boiling buffer for 1 min and 30 s, then allowed to cool naturally. The sections were washed three times with TBST for 5 min each.

Following antigen retrieval, the tissue sections were marked using a demarcating pen and incubated in TBST. Blocking was performed by applying 10% serum (matching the secondary antibody source) and incubating at 37 °C for 30 min. For primary antibody incubation, the serum was removed, and the primary antibody was diluted in 10% serum and applied to the tissue at 50–100 µL per slide, followed by incubation overnight at 4 °C. The sections were then washed three times with TBST, followed by incubation with a secondary antibody diluted in TBST at 50–100 µL per slide, and incubated at 37 °C for 45 min. After washing again three times with TBST, nuclear staining was performed using DAPI (1:500 dilution) for 5 min in the dark, followed by washing with TBST. Finally, the sections were mounted with a fluorescence mounting medium and stored at 4 °C in the dark. Fluorescence imaging was conducted using an Olympus BX53 fluorescence microscope, and images were captured and analyzed accordingly. Image analysis was performed using ImageJ (v2.1.0/1.53c) with the following steps: For RGB images, separate the individual channels (Image > Color > Split Channels). Adjust the image threshold (Image > Adjust > Threshold), selecting the default threshold. The region of interest (ROI) is marked in red, and the “Dark Background” option is enabled to accommodate fluorescence images with dark backgrounds. In the “Analyze” menu (Analyze > Set Measurements), select both “Mean gray value” and “Limit to threshold.” Perform measurement using “Analyze > Measure,” and the “Mean” value in the results corresponds to the average fluorescence intensity for each sample (LGR5 Polyclonal Antibody: Thermo Fisher Scientific Cat# PA5-116472, RRID: AB_2901104, 1:200 dilution).

### Metagenomics

After 72 h of irradiation, four mice per group in the 12 Gy group were euthanized. The intestines were carefully extracted using sterile forceps, and the contents were gently expelled before placing the tissue into a cold container. Samples were then rapidly frozen in liquid nitrogen and stored on dry ice for subsequent analysis. Metagenomic analysis employed the Illumina NovaSeq or HiSeq platform utilizing a Whole Genome Shotgun strategy. Libraries were constructed from extracted metagenomic DNA or synthesized cDNA and subjected to paired-end sequencing. Raw paired-end reads obtained from high-throughput sequencing underwent quality control to remove non-target sequences, resulting in a high-quality dataset suitable for downstream metagenomic analysis. The high-quality reads were corrected and assembled into contigs, followed by redundancy removal and gene prediction to generate a non-redundant set of amino acid sequences. Species annotation was performed using Kaiju, producing abundance tables across six taxonomic levels from domain to species. Taxonomic classification of high-quality reads was conducted using the MMseqs2 taxonomy module against the NCBI nr database (v2021.10.11), applying the lowest common ancestor algorithm for precise species assignment. Functional annotation involved aligning protein sequences with the KEGG, EggNOG, and GO databases using the MMseqs2 search module. Taxonomic composition analysis was performed with QIIME to generate abundance distribution tables, and results were visualized by mapping to the NCBI Taxonomy tree using MEGAN. Alpha diversity was quantified using the Chao1, ACE, Shannon, and Simpson indices, while beta diversity was assessed through principal component analysis (PCA). Linear discriminant analysis effect size (LEfSe) was used to identify significantly enriched taxa between groups by combining the Kruskal-Wallis test and linear discriminant analysis (LDA). The Kruskal-Wallis test assessed the statistical significance of abundance differences across groups, while LDA was applied to estimate the effect size of each taxon. A threshold LDA score of 2.0 was used to define significantly different taxa, which were considered as potential biomarkers. The results were visualized using R packages, with LDA scores displayed in bar plots to highlight the most significant taxa, and a cladogram was used to illustrate the taxonomic distribution of biomarkers across samples.

### Non-targeted metabolism

72 h after irradiation, five mice from each 12 Gy group were euthanized, and a segment of the jejunum was collected from each mouse. The intestinal tissue samples were rinsed with physiological saline, cut into small pieces, and placed in cryovials. The samples were then frozen in liquid nitrogen, stored on dry ice, and sent to the testing laboratory. An appropriate amount of sample was accurately weighed and placed into a centrifuge tube. Tissue extract and steel beads were added, and the sample was then ground. After ultrasonic treatment, the sample was centrifuged, and the supernatant was collected in a new tube, concentrated, and dried. Next, a 50% acetonitrile solution containing 2-chloro-L-phenylalanine was added to re-dissolve the sample. After filtration, the samples were transferred into detection vials for LC-MS analysis. The samples were separated using a liquid chromatography system (Thermo Vanquish), with components sequentially entering the mass spectrometer (Thermo Orbitrap Exploris 120) for continuous scanning and data acquisition. The raw data were initially converted to mzXML format using the MSConvert tool in the ProteoWizard software package (v3.0.8789) [30] and processed with the R XCMS package (v3.12.0) for feature detection [31], retention time correction, and alignment. Key parameter settings included: ppm = 15, peakwidth = c (5, 30), mzdiff = 0.01, and method = centWave. The data were then corrected using area normalization to eliminate systematic errors.

Metabolites were identified based on accurate mass and MS/MS data, which were compared against public databases such as HMDB, MassBank, KEGG, LipidMaps, and mzCloud [32–36], as well as an in-house metabolite database developed by Panomix Biomedical Tech Co., Ltd. (Suzhou, China). The molecular weight of metabolites was determined from the m/z (mass-to-charge ratio) of parent ions in the MS data, and the molecular formula was predicted using ppm (parts per million) and adduct ions. The predicted formula was then matched with the database for primary MS identification. Additionally, MS/MS data from the quantitative table were matched with fragment ions and other metabolite information in the database to enable secondary MS/MS identification.

Two multivariate statistical models, unsupervised and supervised (PCA, PLS-DA, OPLS-DA), were applied to discriminate between groups using the R ropls package (v1.22.0) [37]. Statistical significance was assessed using *p*-values from group comparisons. Biomarker metabolites were selected based on *p* -value, VIP (variable importance in projection from OPLS-DA, and fold change (FC). By default, metabolites with *p* -value < 0.05 and VIP >1 were considered to have significant differential expression. KEGG pathway enrichment analysis is typically conducted using the MetaboAnalyst 5.0 [38], based on the KEGG database. The *p*-values are calculated using the hypergeometric test, and multiple hypothesis correction is performed using the Benjamini-Hochberg method. Pathway selection was primarily based on an FDR threshold of < 0.05; however, due to the absence of significant pathways, *p* < 0.05 was used for exploratory pathway identification in this study. The identified metabolites were mapped to the KEGG pathways for biological interpretation of systemic functions, and the results were visualized using the KEGG Mapper tool.

### Single-cell sequencing

72 h post-irradiation, one mouse from each group was euthanized to collect the jejunum. The tissue was washed with saline, cut into small pieces, and immediately placed in single-cell preservation solution before being transferred to the laboratory under low temperature conditions. After washing with phosphate-buffered saline, enzymatic digestion was performed to obtain a single-cell suspension. Single-cell RNA sequencing was performed using the 10X Genomics Chromium platform (10X Genomics, Pleasanton, CA). The samples were subjected to quality control according to single-cell suspension standards. The prepared cell (nuclei) suspension, 10X barcode gel beads, and oil were separately added to the individual chambers of the Chromium Chip G to form Gel Beads in Emulsion, followed by RNA sequencing and reverse transcription. Following cDNA amplification, the cDNA library was constructed. Cluster generation and first-strand sequencing primer hybridization were performed according to the Illumina User Guide. The flow cell containing the clusters was then loaded onto the sequencer for paired-end sequencing. The sequencing process was controlled by the Illumina data collection software, with real-time data analysis performed. The data were first processed using the CellRanger v7.0.1from 10X Genomics for alignment, gene quantification, and cell identification. Subsequently, the data were further analyzed and filtered using Seurat v3.0.2, including cell subpopulation analysis, GO/KEGG enrichment analysis, and cell communication analysis. Specifically, CellRanger was employed to convert the raw fastq files into a gene expression matrix [39]. Based on the distribution of UMI counts, gene expression, mitochondrial genes, and ribosomal genes, filtering thresholds were set for data preprocessing and normalization [40]. Next, Louvain clustering (resolution = 0.5) was applied to the normalized data, followed by dimensionality reduction using PCA, t-SNE, or UMAP for visualization of the clustering results. The Wilcoxon algorithm was used to identify marker genes for each cluster, and a “group one vs. rest” approach was employed to score these genes. Genes with a logFC >0.25 and expression in at least 20% of the cells were selected as significant marker genes. Further, KEGG and GO functional enrichment analyses were performed to infer the functional characteristics of each cluster based on the pathways and GO terms associated with the marker genes. singleR v1.0.0 [41] and SCINAv1.2.0 [42] were utilized for automated and semi-supervised cell type annotation, respectively. Cell activity levels were calculated using the ssGSEA algorithm from the GSVA package and visualized with ggplot2. Finally, CellChat v1.1.0 was applied to analyze ligand-receptor pairs, further inferring cell-to-cell signaling pathways and communication networks [43].

### Statistical analysis

Statistical analyses were performed using GraphPad Prism 9.0 (RRID: SCR_002798) and R software 4.2.2 (RRID: SCR_001905). Comparisons between two groups were conducted using unpaired two-tailed Student’s t-tests, with effect sizes calculated using Cohen’s d for pairwise comparisons. Comparisons among multiple groups were performed using one-way analysis of variance (ANOVA) followed by Tukey’s post hoc test, with effect sizes quantified using eta squared (η²) for ANOVA. Survival curves were generated using the Kaplan–Meier method, and differences between groups were assessed using the log-rank test. All statistical tests were conducted with adjustments for multiple comparisons, using the Benjamini–Hochberg method for omics data, and Bonferroni correction for pairwise comparisons in t-tests and ANOVA. Adjusted *p*-values (adj.*p*) < 0.05 were considered statistically significant. *p* < 0.05 was considered statistically significant. For omics data—including metagenomic, untargeted metabolomic, and single-cell RNA sequencing—statistical computations and visualizations were carried out in R using dedicated packages such as Seurat, vegan, XCMS, and MetaboAnalystR. In metagenomic analyses, intergroup differences were assessed using Adonis (PERMANOVA) and ANOSIM tests, and differential taxonomic abundance was identified using the metagenomeSeq algorithm. Relevant multiple hypothesis testing was adjusted using the Benjamini–Hochberg method, and adjusted *p*-values (adj.*p*) < 0.05 were considered statistically significant.The Benjamini-Hochberg FDR correction was systematically applied to relevant high-dimensional analyses, including species differential analysis in metagenomics, pathway enrichment analysis in untargeted metabolomics, and intergroup differences and enrichment analysis in single-cell sequencing. “*” denotes *p* < 0.05; “” denotes *p* < 0.01; “” denotes *p* < 0.001; “**” denotes *p* < 0.0001.

## Results

### FLASH-RT achieved antitumor efficacy comparable to that of CONV-RT

**Fig. 2:**
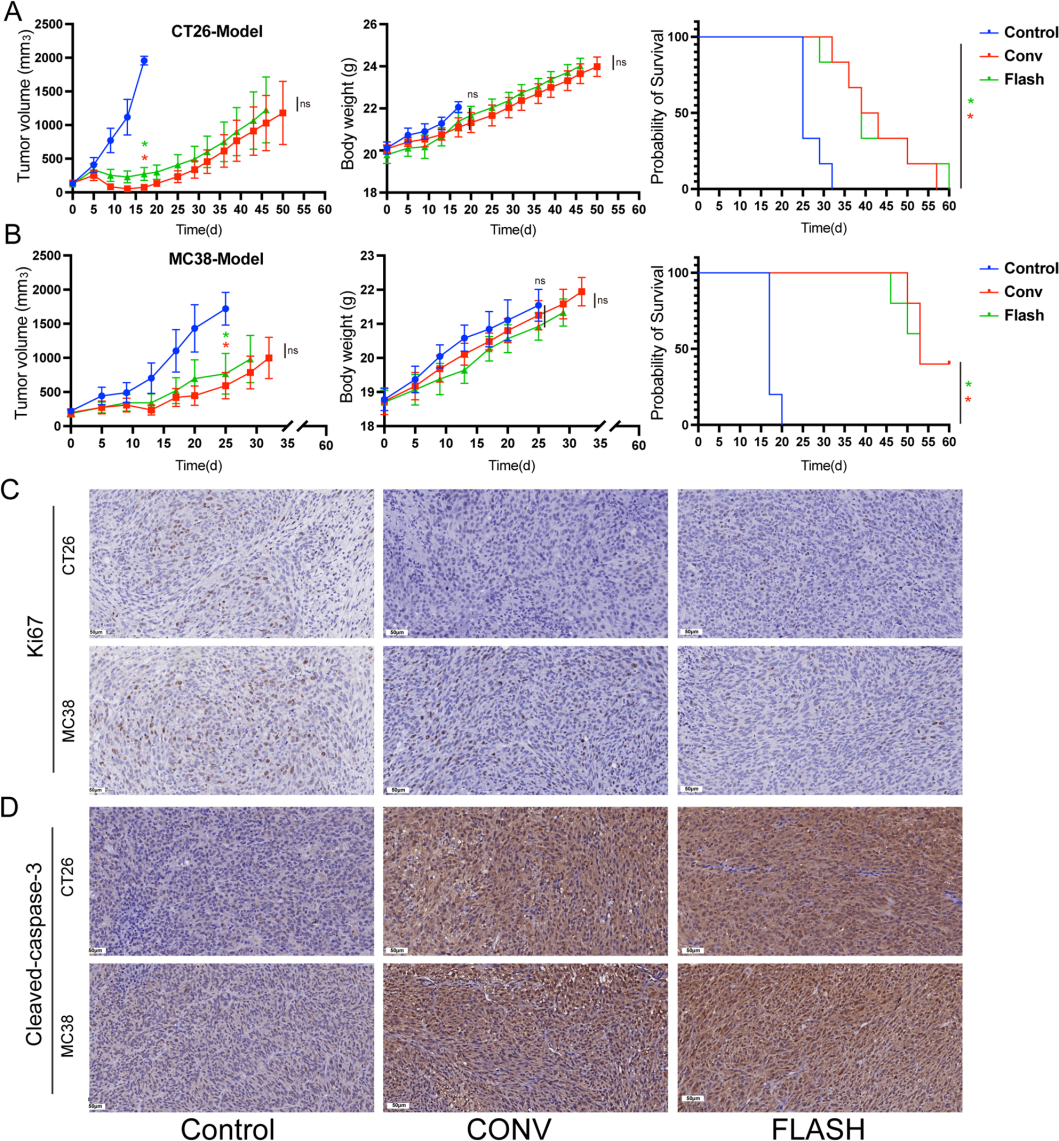
FLASH-RT exhibits antitumor effects comparable to those of CONV-RT. (**A**) Tumor growth curves, average body weight curves, and survival curves for the CT26 tumor model. (**B**) Tumor growth curves, average body weight curves, and survival curves for the MC38 tumor model. Tumor growth curves were censored for a group when its average volume reached the predefined ethical endpoint. Only groups not meeting the endpoint are shown thereafter. Subsequent outcomes for all groups are presented in the survival curves. Data for tumor volume and body weight were analyzed using two-way ANOVA followed by Tukey’s post hoc test. Effect sizes for the two-way ANOVA (η²) for tumor volume and body weight analyses are reported in the results section. Pairwise comparisons of tumor volume was further analyzed using Cohen’s d to quantify the effect sizes for each comparison. For survival analysis, the Log-rank test was used, and statistical significance was determined based on *p*-values. Statistical significance was determined as *p* < 0.05 (denoted as *). (**C**) Representative immunohistochemical images of Ki67 expression in tumors from each tumor model. (**D**) Representative immunohistochemical images of Cleaved Caspase-3 expression in tumors from each tumor model

The antitumor efficacy of CHEXs was evaluated using subcutaneous CT26 and MC38 tumor models. Tumor growth inhibition was assessed in both models when control mice reached the ethical endpoint. In the CT26 model, both CONV and FLASH treatment significantly suppressed tumor growth compared with the control group (114.78 ± 51.79 mm³ vs. 226.06 ± 217.96 mm³ vs. 1907.05 ± 144.57 mm³; *p* < 0.05, η² = 0.6055), with no significant difference observed between the CONV and FLASH groups (*p* > 0.05, Cohen’s d = 2.85, Fig. 2A). No significant differences in body weight were detected among treatment groups in the CT26 model (η² = 0.0675, *p* > 0.05). Similarly, in the MC38 model, both CONV and FLASH treatment markedly inhibited tumor growth compared with the control group (593.67 ± 471.56 mm³ vs. 717.94 ± 662.15 mm³ vs. 1719.22 ± 539.21 mm³; *p* < 0.05, η² = 0.1411), with no significant difference in tumor volume between the CONV and FLASH groups (*p* > 0.05, Cohen’s d = 0.430, Fig. 2B). Tumor volume curves for individual mice are shown in Supplementary Fig.S1. No statistically significant difference in body weight was observed among the MC38 treatment groups, with η² = 0.0381. Survival times of tumor-bearing mice in the CONV and FLASH groups were significantly longer than those in the control group for both models (*p* < 0.05). Immunohistochemical analysis revealed that Ki67 staining was significantly lower in the FLASH and CONV groups compared to the control group, with no significant difference between the two treatments (Fig. 2C). Additionally, Cleaved Caspase-3 staining was significantly higher in the FLASH and CONV groups than in the control group, with no difference between the treatment groups (Fig. 2D).

### FLASH-RT demonstrates a protective effect on normal intestinal tissues

**Fig. 3:**
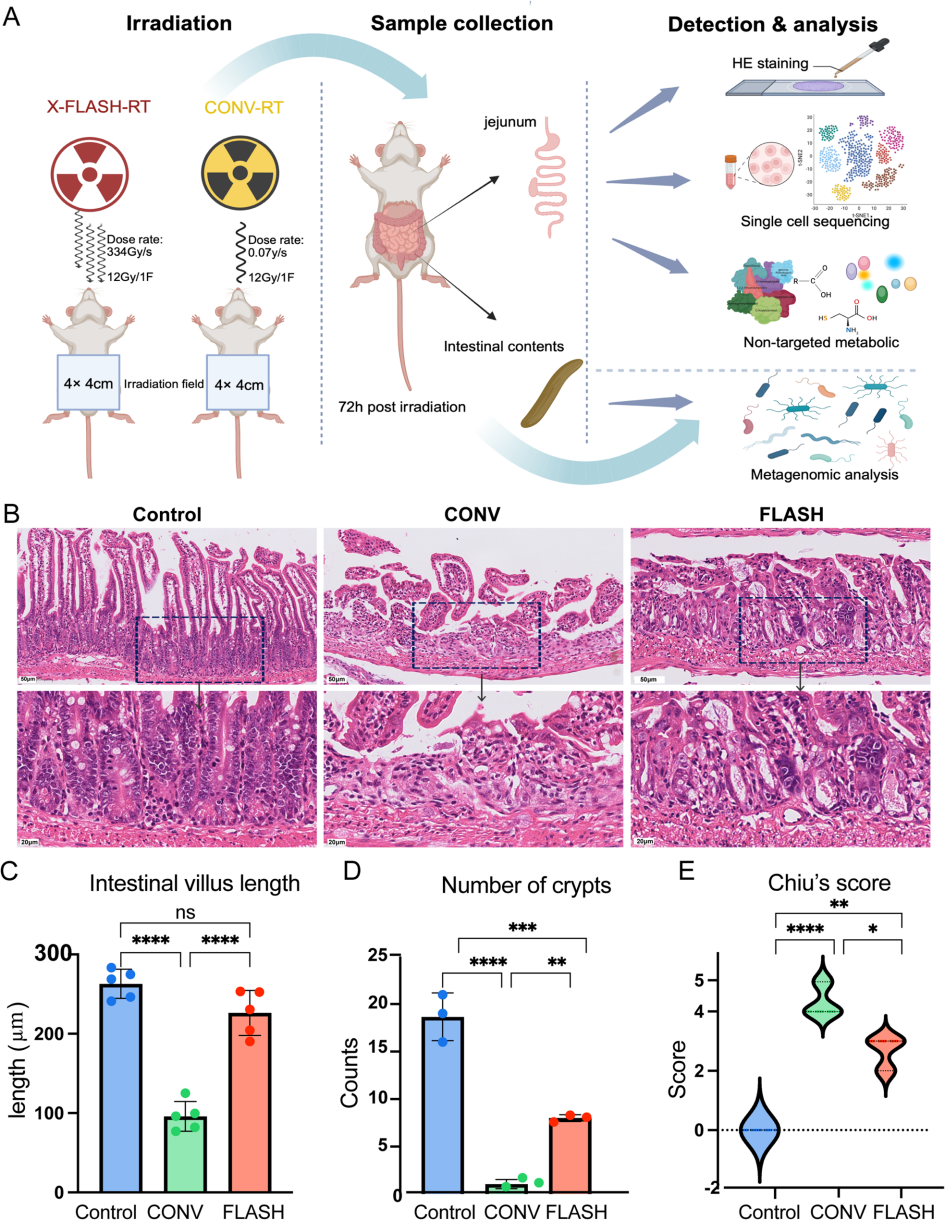
FLASH-RT alleviates damage to normal intestinal tissue compared to CONV-RT. (**A**) Schematic diagram of sample collection and analysis. (**B**) Representative H&E staining images of each group 72 h post-abdominal irradiation. (**C**) Comparison of villus height in the small intestine of each group 72 h post-abdominal irradiation. (**D**) Comparison of the number of crypts regenerating per millimeter in each group 72 h post-abdominal irradiation. (**E**) Chiu’s scores of intestinal tissue damage in each group 72 h post-abdominal irradiation. Data were analyzed using one-way ANOVA followed by Tukey’s post hoc test. Pairwise comparisons of villus length, crypts per millimeter and Chiu’s score were further analyzed using Cohen’s d to quantify the effect sizes for each comparison. Statistical significance was determined as *p < 0.05, **p < 0.01, *** *p* < 0.001, and ****p < 0.0001

The sampling and analysis diagram for each omics group is shown in Fig. 3A. Histological analysis of intestinal tissue on day 3 post-whole abdomen irradiation revealed significant differences between the treatment groups. In the CONV group, extensive epithelial necrosis, detachment, and ulceration were observed, along with loss of villi and crypt structure, accompanied by pronounced inflammatory cell infiltration. In the FLASH group, the extent of epithelial necrosis and detachment was less severe, with some villus atrophy, shortening, and crypt damage, accompanied by mild inflammatory cell infiltration. Overall, compared to the CONV group, the FLASH group exhibited less inflammation and damage (Fig. 3B). The villus length in the control group was greater than that in the FLASH and CONV groups. Statistically significant differences in villus length were observed between the FLASH and CONV groups (*p* < 0.0001, Cohen’s d = 9.24, Fig. 3C). The number of intestinal crypts per millimeter in the control group was significantly higher than in the FLASH and CONV groups (*p* < 0.0001). The FLASH group showed a significantly higher number of crypts per millimeter than the CONV group (*p* < 0.01, Cohen’s d = 5.72, Fig. 3D). Chiu’s score for the different groups indicated that intestinal damage in the CONV group was more severe than in the FLASH and control groups. Statistically significant differences in intestinal damage scores were observed between the CONV and FLASH groups (*p* < 0.05, Cohen’s d = 6.92, Fig. 3E). Given that the protective effect of FLASH-RT has been confirmed in intestinal tissues, we next performed multi-omics analysis on mice following 12 Gy whole-abdomen irradiation.

### Integrated metagenomic and metabolomic analyses implicate a candidate microbiota-metabolite axis in FLASH-RT-mediated tissue protection

To investigate the potential role of the gut microbiome and its functional metabolites in FLASH-RT-mediated tissue protection, we performed metagenomic sequencing on fecal samples collected 72 h post-irradiation and conducted untargeted metabolomics on intestinal tissue.

**Fig. 4:**
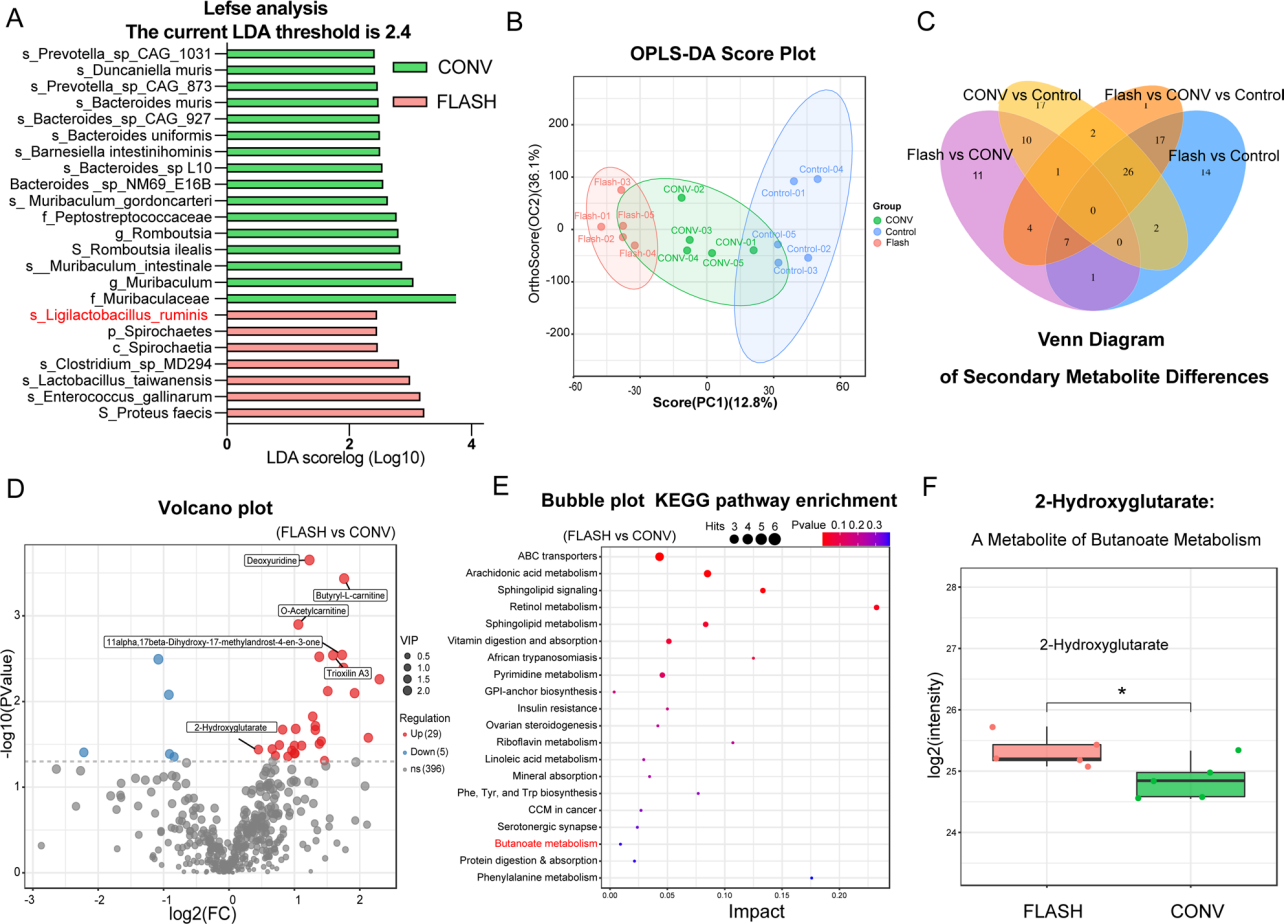
Metagenomic and non-targeted metabolomic analyses reveal enrichment of *Ligilactobacillus ruminis* and butanoate metabolism related metabolites in the FLASH group. (**A**) The LEfSe species bar chart shows significantly different species between the FLASH and CONV groups (LDA score > 2.4, p < 0.05). (**B**) OPLS-DA score plots for each group. (**C**) Venn diagram of secondary metabolites. (**D**) Volcano plot illustrating differential metabolite expression between the FLASH group and CONV group. Axes: The x-axis represents the log2 transformed fold change (Log2 Fold Change) in metabolite abundance between groups; the y-axis denotes the log10 transformed p value (statistical significance, with higher values indicating stronger significance). (**E**) Bubble plot of KEGG metabolic pathway enrichment (FLASH vs. CONV). The x-axis denotes the pathway impact score (a composite metric integrating the number and fold changes of differential metabolites). The y-axis lists enriched metabolic pathways. Bubble size: Reflects the number of differential metabolites (hits) in each pathway. Color gradient (red to blue): Represents statistical significance (p-value), with red indicating more significant enrichment. (**F**) Box plot showing the abundance of 2-hydroxyglutarate across experimental groups. The differences between the two groups were analyzed using an unpaired t-test. The effect size is presented using Eta squared (η²)

Metagenomic analysis revealed alterations in microbial community structure at the species level among the FLASH, CONV, and control groups. Linear discriminant analysis Effect Size (LEfSe) identified taxa with differential enrichment between the FLASH and CONV groups. Specifically, in the FLASH group, the relative abundances of *Ligilactobacillus ruminis* (LDA = 2.47, *p* = 0.04), *Lactobacillus taiwanensis* (LDA = 3.01, *p* = 0.02), *Enterococcus gallinarum* (LDA = 3.17, *p* = 0.04), and *Proteus faecis* (LDA = 3.24, *p* = 0.02) were significantly enriched. In contrast, in the CONV group, *Peptostreptococcaceae* (LDA = 2.79, *p* = 0.02), *Romboutsia ilealis* (LDA = 2.85, *p* = 0.02), *Bacteroides muris* (LDA = 2.49, *p* = 0.04), and *Muribaculum* (LDA = 3.42, *p* = 0.02) represented the predominant taxa (Fig. 4A). Based on the literature review, we focused on Ligilactobacillus ruminis, a species reported to produce short-chain fatty acids (SCFAs), particularly butyrate, which has been shown to reduce pro-inflammatory cytokines, alleviate colitis, and promote epithelial repair [44, 45]. Its significant enrichment in the FLASH group suggests a potential correlation with the reduced intestinal injury following FLASH irradiation.

We next analyzed the intestinal tissue metabolome to identify functional metabolic correlates of the microbial changes. PCA and OPLS-DA analyses revealed clear separation between groups, confirming distinct metabolic states (Fig. 4B, S2). Permutation tests validated the robustness and statistical significance of the OPLS-DA model, ruling out overfitting (Fig. S3). High model fitting and predictability (e.g., CONV vs. FLASH: R²Y = 0.996, Q² = 0.528) further confirmed the validity of the analysis.

Differential metabolites were selected based on a univariate *p*-value (*p* < 0.05) from Student’s t-test and a multivariate variable importance in projection (VIP > 1.0) from the OPLS-DA model. A total of 430 differential secondary metabolites were identified. A Venn diagram illustrated the unique and overlapping metabolites among groups, showing 11 unique metabolites in the FLASH versus CONV comparison, 10 in CONV versus control, and 14 in FLASH versus control (Fig. 4C). In the FLASH vs. CONV comparison, 34 differentially expressed metabolites were identified, with 29 upregulated and 5 downregulated in the FLASH group. The top five differential metabolites, ranked by the magnitude of change (|log₂FC|) and VIP score, were 1,2,3-Trihydroxybenzene (VIP = 1.79; log_2_FC = 1.01), Pipecolic acid (VIP = 1.88; log_2_FC = 0.66), O-Phosphoethanolamine (VIP = 1.68; log_2_FC = 1.46), Hydroxyphenyllactic acid (VIP = 2.14; log₂FC = 0.92), and O-Acetylcarnitine (VIP = 2.33; log_2_FC = 1.06) (Fig. 4D).

KEGG pathway enrichment analysis of the differential metabolites was performed using MetaboAnalyst via a hypergeometric test. While no pathways remained significant after FDR correction, the top enriched pathways based on a nominal *p* < 0.05 were selected. The most significantly enriched pathways in the FLASH group included ABC transporters (*p* = 0.0018; Impact = 0.043), Arachidonic acid metabolism (*p* = 0.0065; Impact = 0.085), Sphingolipid signaling pathway (*p* = 0.0087; Impact = 0.133), Retinol metabolism (*p* = 0.024; Impact = 0.232), and Sphingolipid metabolism (*p* = 0.044; Impact = 0.083). Butanoate metabolism was also identified (*p* = 0.36, Impact = 0.009, Fig. 4E).

Based on these results and supporting literature, we specifically focused on the butyrate metabolism pathway. Butyrate, a key SCFA produced by gut microbes such as *L. ruminis*, is known to promote intestinal epithelial renewal and repair [46], inhibit the production of pro-inflammatory cytokines via HDAC inhibition and suppression of NF-κB [47], and maintain gut homeostasis [48, 49]. Consistent with the metagenomic findings, further analysis revealed that 2-hydroxyglutarate, a metabolite associated with the butyrate pathway, was significantly increased in the FLASH group compared to the CONV group (Fig. 4F, *p* = 0.045, η² = 0.41). Other metabolites related to butyrate metabolism, including (R)-3-Hydroxybutyric acid, Oxoglutaric acid, Fumaric acid, and L-Glutamic acid, also exhibited higher expression levels in the FLASH group (Fig. S4). This integrated multi-omics insight suggests that the enrichment of beneficial butyrate-producing bacteria and the subsequent modulation of related metabolic pathways may be associated with the intestinal protection conferred by FLASH-RT.

### Single-cell sequencing and tissue staining reveal enhanced regenerative potential and reduced inflammation in FLASH-irradiated intestine

**Fig. 5:**
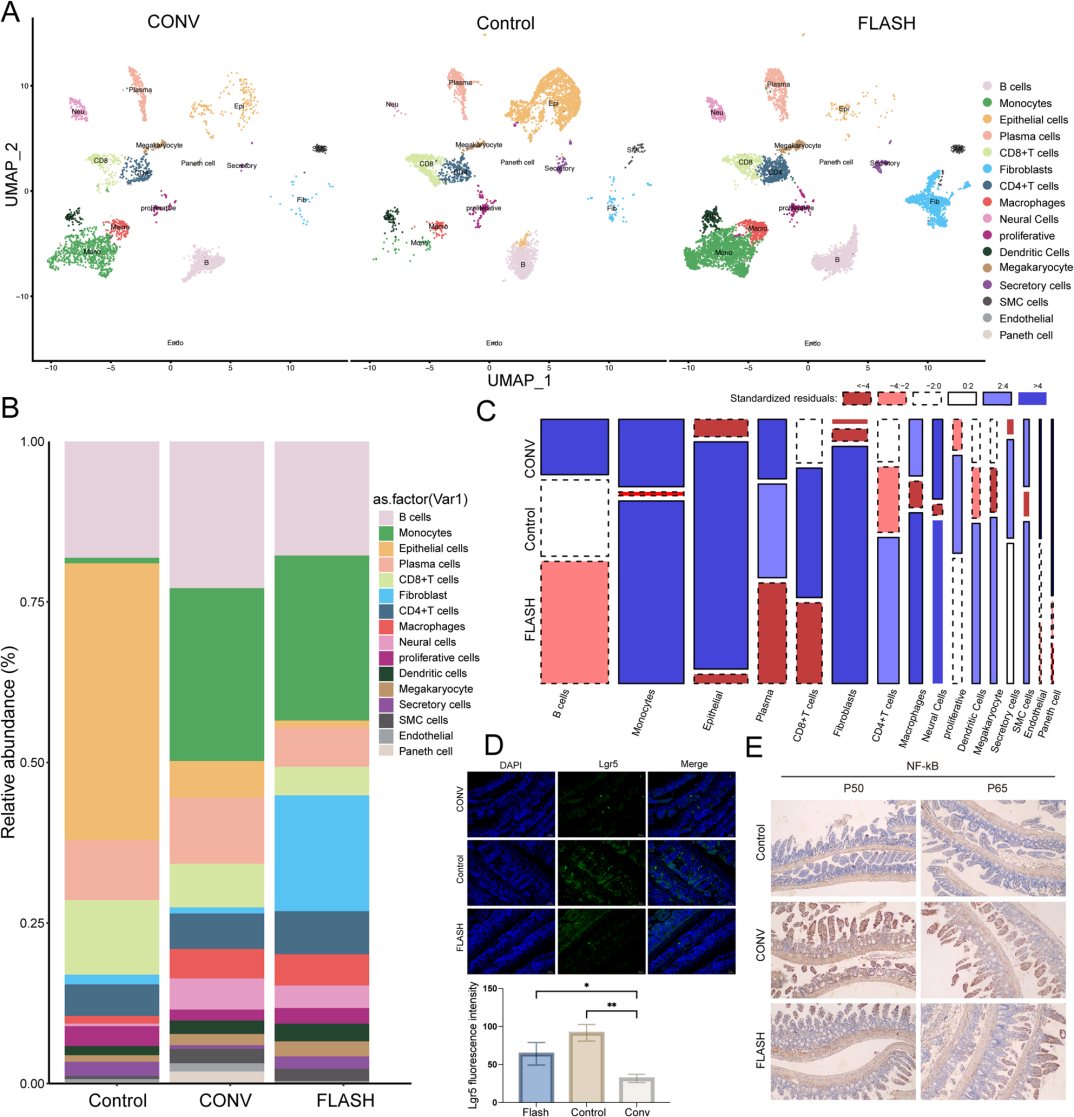
FLASH-RT promotes intestinal stem cell maintenance and reduces NF-κB activation while altering cellular composition in the irradiated intestine. (**A**) UMAP visualization of clusters for each group. (**B**) Relative abundance of each cell type across the groups. (**C**) Proportion of each cluster in different groups. (**D**) Representative Lgr5 immunofluorescence images and corresponding fluorescence intensity bar graph for each group. Data were analyzed using one-way ANOVA followed by Tukey’s post hoc test. Effect sizes were quantified using eta squared (η²) for ANOVA. Pairwise comparisons of Lgr5 average fluorescence intensity was further analyzed using Cohen’s d to quantify the effect sizes for each comparison. “*” denotes *p* < 0.05; “**” denotes *p* < 0.01; “***” denotes *p* < 0.001. (**E**) Representative p50 and p65 immunohistochemistry images for each group

After quality control filtering, 3,325 cells from the control group, 7,368 cells from the CONV group, and 9,125 cells from the FLASH group were retained for subsequent analyses. To characterize the cellular composition of the intestinal tissue, we leveraged single cell RNA sequencing data and known marker genes (Fig.S5) to perform cell type annotation. Through unsupervised clustering and validation of marker gene expression, we identified 16 distinct cellular subpopulations, including epithelial, immune, and stromal cells. Marker genes for each cluster were identified using the Wilcoxon rank-sum test with a “one-versus-rest” strategy, and their expression changes were quantified by log_2_ Fold Change. The distribution of cell populations in low-dimensional space was visualized using UMAP (Fig. 5A). Cluster analysis revealed differences in the proportion of cells across various samples (Fig. 5B and C, Fig. S6). The results indicated that the proportion of fibroblasts, proliferative cells, Macrophages, and CD4 + T cells in the FLASH group was higher than that in the CONV group. To further elucidate the impact of FLASH-RT on stem cells involved in intestinal epithelial regeneration and on intestinal inflammation, intestinal tissues collected 72 h after irradiation were subjected to immunohistochemistry (IHC) and immunofluorescence analyses. Immunofluorescence results revealed a significantly higher intensity of Lgr5 staining in the FLASH group compared to the CONV group (*p* = 0.0014, Fig. 5D), indicating a greater proportion of intestinal stem cells (the primary proliferative cell population in the intestine) in the FLASH-RT tissues. IHC analysis of key NF-κB subunits demonstrated that the staining intensities of p50 and p65 were lower in the FLASH group than in the CONV group (Fig. 5E), suggesting an attenuated inflammatory response in the FLASH group.

To elucidate the potential protective mechanisms of FLASH-RT compared to CONV-RT in normal intestinal tissue, we performed Gene Set Variation Analysis (GSVA) using the ssGSEA algorithm. This analysis systematically compared differences in KEGG pathway activation among key intestinal cell populations—such as epithelial cells, proliferative cells, fibroblasts, and macrophages—across the Control, FLASH, and CONV groups.

**Fig. 6:**
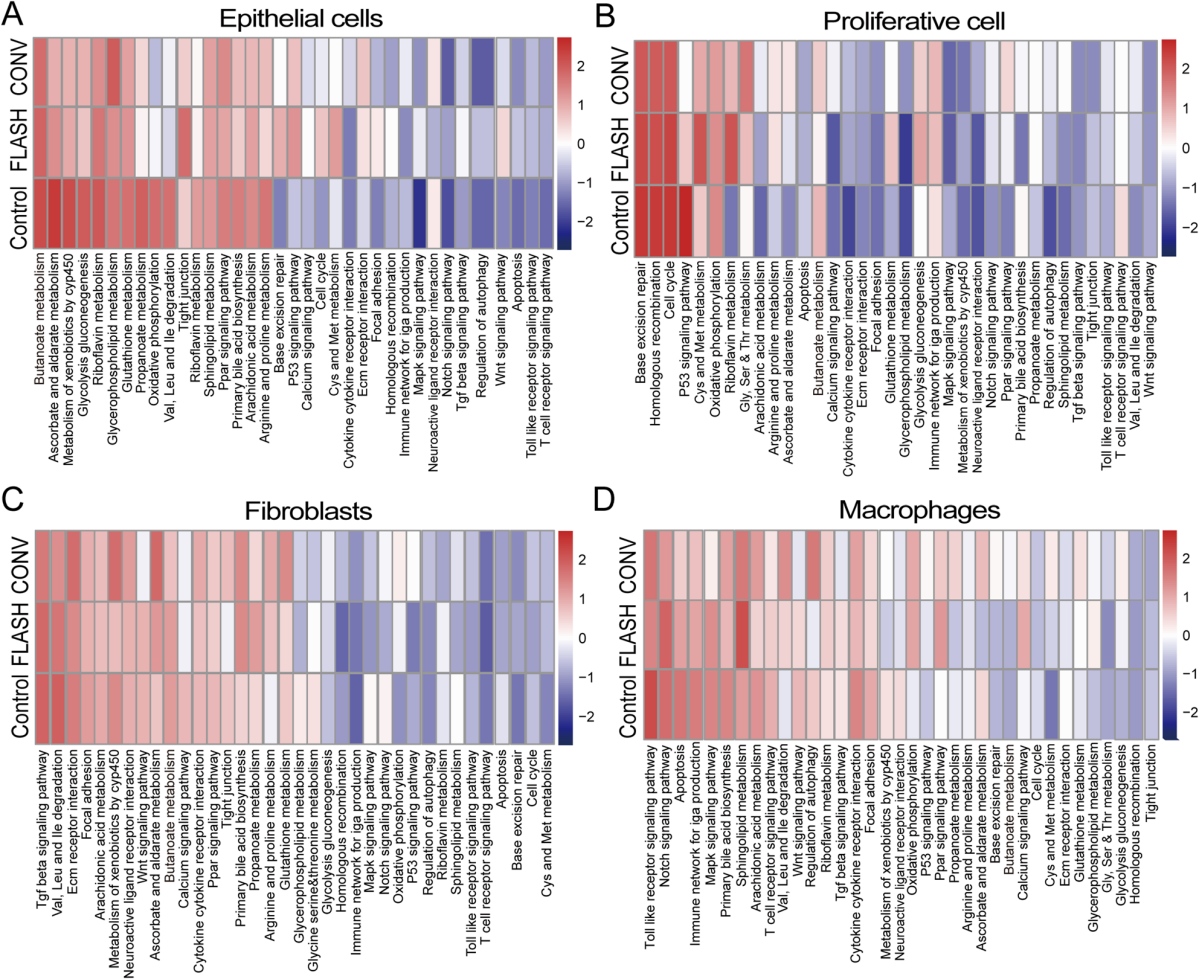
Altered pathway activities across major intestinal cell types following irradiation. Heatmap depicting Gene Set Variation Analysis (GSVA) scores for selected KEGG pathways in epithelial cells (**A**), proliferative cells (**B**), fibroblasts (**C**), and macrophages (**D**) from control, FLASH-RT, and CONV-RT groups. Each row represents a pathway; each column represents a cell type from a treatment group. The color scale represents the GSVA enrichment score, with red indicating upregulation and blue indicating downregulation of the pathway

As the core of the intestinal physical and functional barrier, epithelial cells exhibited extensive pathway activation following FLASH-RT. Key pathways, including Wnt signaling, tight junctions, glycine/serine/threonine metabolism, p53 signaling, cell cycle, cysteine and methionine metabolism, ECM-receptor interactions, focal adhesion, homologous recombination, MAPK signaling, and TGF-β signaling, all showed increased GSVA scores. In contrast, the CONV-RT group mainly activated glycerophospholipid metabolism and PPAR signaling pathways. These results suggest that FLASH-RT contributes to maintaining epithelial proliferation and regeneration, stabilizing barrier structures, and promoting efficient DNA damage repair (Fig. 6A).

In the proliferative cell population, rich in intestinal stem cells, FLASH-RT showed significant activation of pathways related to base excision repair, cysteine and methionine metabolism, riboflavin metabolism, glutathione metabolism, p53 signaling, and glycolysis/gluconeogenesis. On the other hand, the CONV-RT group primarily activated pathways associated with glycine/serine/threonine metabolism and calcium signaling. This pattern indicates that FLASH-RT might support rapid cellular repair and regeneration after radiation-induced damage by enhancing DNA damage response, redox metabolism, and energy supply (Fig. 6B).

Analysis of fibroblasts in the stromal microenvironment revealed higher pathway activity in FLASH-RT for Wnt signaling, butyrate metabolism, glycine/serine/threonine metabolism, riboflavin metabolism, base excision repair, and propionate metabolism. The CONV-RT group primarily activated ECM-receptor interactions, cytochrome P450 xenobiotic metabolism, ascorbate and aldarate metabolism, glutathione metabolism, Notch signaling, and oxidative phosphorylation (Fig. 6C). In macrophages, FLASH-RT activated Notch signaling, MAPK signaling, sphingolipid metabolism, and PPAR signaling pathways. Conversely, CONV-RT enriched several pathways, including amino acid metabolism, branched-chain amino acid degradation, autophagy, cytokine-cytokine receptor interactions, propionate metabolism, ascorbate and aldarate metabolism, and glutathione metabolism (Fig. 6D).

## Discussion

This study validated the FLASH effect of high-energy X-ray FLASH-RT and preliminarily explored mechanisms of normal intestinal tissue protection via multi-omics analysis. The results demonstrated that FLASH-RT achieved tumor suppression comparable to CONV-RT in both the MC38 and CT26 models.HE of intestinal tissue revealed less severe damage in the FLASH group, confirming the dual advantage of FLASH-RT in preserving antitumor efficacy while reducing radiotoxicity to intestinal tissue, consistent with previous reports [23, 50].

Mechanistically, metagenomic analysis indicated an increased relative abundance of beneficial bacteria such as Ligilactobacillus ruminis following FLASH-RT. Such bacteria can produce SCFAs, particularly butyrate, through dietary fiber fermentation. Butyrate plays a key role in maintaining intestinal barrier integrity, suppressing inflammation, and promoting epithelial repair [45, 48, 51–53]. Although untargeted metabolomics did not show statistically significant enrichment of butyrate metabolism pathways in intestinal tissues, the content of 2-hydroxyglutarate was significantly elevated in the FLASH group, and several other butyrate-related metabolites also showed increasing trends, suggesting a potential involvement of butyrate metabolism in the FLASH effect. This finding is corroborated by single-cell transcriptomic data showing activation of the butyrate metabolism pathway in fibroblasts from the FLASH group. Further validation via immunofluorescence and immunohistochemical analysis showed that the FLASH group had a stronger Lgr5 + stem cell signal and lower expression of the NF-κB subunits p50/p65 than the CONV group, indicating greater stem cell preservation and inhibited inflammatory activation by FLASH-RT [54]. Together, these results suggest that FLASH-RT may exert intestinal protection indirectly by modulating the microbiota-metabolite axis, especially butyrate metabolism. This hypothesis aligns with established mechanisms whereby butyrate promotes stem cell proliferation via Wnt/β-catenin signaling [46], enhances the epithelial barrier via AMPK signaling [55], and alleviates inflammation through inhibition of the NF-κB pathway [47].

At the cellular level, GSVA based on single-cell transcriptomics revealed a multicellular cooperative network underpinning the protective effects of FLASH-RT. In intestinal epithelial cells, FLASH-RT specifically activated pathways directly related to epithelial barrier structure, such as tight junctions, ECM-receptor interaction, and focal adhesion, suggesting active maintenance and repair of the physical barrier function—critical for preventing bacterial translocation and endotoxemia after irradiation [56, 57]. Concurrent activation of Wnt signaling and cell cycle pathways indicated preserved proliferative and self-renewal potential [58, 59], consistent with the increased Lgr5 + stem cells observed via immunohistochemistry.

In proliferative cells (mainly intestinal stem cells) [60, 61], both FLASH-RT and CONV-RT activated DNA damage repair pathways such as base excision repair and homologous recombination, indicating comparable induction of DNA damage responses [62, 63]. In contrast, CONV-RT activated calcium signaling and apoptotic pathways, driving cells toward irreversible death [64, 65]. This difference may explain the superior preservation of proliferative cells and maintenance of intestinal regenerative capacity following FLASH-RT. In macrophages, FLASH-RT induced notable immunomodulatory effects: significant activation of the PPAR signaling pathway, a key driver steering macrophages toward an anti-inflammatory/reparative (M2-like) phenotype [66], likely contributing to reduced proinflammatory cytokine release via suppression of NF-κB pathway [67]. This aligns with our observation of lower p50 and p65 staining intensity and attenuated inflammatory response in the FLASH group. This contrasts sharply with the activation of classical pro-inflammatory (M1-like) pathways, such as arachidonic acid metabolism, which synthesizes pro-inflammatory mediators, observed in macrophages from the CONV-RT group [68]. These findings suggest that FLASH-RT may reprogram the tumor immune microenvironment by shifting macrophage phenotype from disruptive to reparative, thus promoting tissue repair.

In fibroblasts, the specificity of FLASH-RT response was evidenced by activation of butyrate metabolism and PPAR signaling pathways. The former echoes the aforementioned microbiota-metabolite axis, while the latter aligns with observations in macrophages, suggesting that PPARγ activation may be a common transcellular feature contributing to an overall anti-inflammatory and anti-fibrotic microenvironment. In contrast, CONV-RT activated the ECM-receptor interaction pathway, indicating fibroblast-to-myofibroblast transition, which may underlie the development of late radiation-induced fibrosis [69].

This study has several limitations. First, although multi-omics data provide strong associative evidence, this remains a correlative study, and functional experiments (e.g., using agonists or inhibitors) are needed to validate causal relationships between the identified pathways and FLASH-mediated protection. Second, discrepancies in sample sources for metagenomics (gut content) and metabolomics (intestinal tissue) may affect metabolite detection and interpretation. Third, while the statistical power of certain omics analyses may be limited by the sample size, the combined multi-omics approach still offers a valuable foundation for future research. Fourth, while the protective effects of FLASH-RT are generally attributed to upstream physicochemical mechanisms (e.g., transient oxygen depletion), this study primarily focused on downstream biological changes including microbiota, metabolic pathways, and inflammatory cytokines; the upstream mechanisms require further exploration. Finally, although multi-omics analyses provide preliminary insights and were partially validated via immunohistochemistry and immunofluorescence, the current findings necessitate replication and validation in additional in vivo and in vitro models.

In summary, this multi-omics study preliminarily revealed that the downstream biological effects of FLASH-RT constitute a multisystem and multicellular cooperative process. The protective effect on normal intestinal tissue may result from the combined contributions of stem cell preservation, suppression of inflammation, and remodeling of the microbiota–metabolite axis. These findings provide new biological insights into the FLASH effect and generate several testable hypotheses. Future studies should employ gene knockout models and pharmacological interventions to functionally validate the potential pathways identified in this study, such as butyrate metabolism, stem cell regulation, and NF-κB signaling, thereby establishing a solid theoretical foundation for clinical translation.

## Conclusion

This study confirms that FLASH-RT, compared to CONV-RT, maintains equivalent antitumor efficacy while mitigating damage to normal intestinal tissues. Moreover, it preliminarily reveals that the protective mechanism of FLASH-RT is multifaceted, involving remodeling of the microbiota-metabolite axis, attenuation of inflammatory responses, and enhanced preservation of stem cells.

## Supplementary Information

Below is the link to the electronic supplementary material.

Supplementary Material 1

## Data Availability

The raw sequence data reported in this paper have been deposited in the Genome Sequence Archive [70] and the multi-omics data archiving database at the National Genomics Data Center [71], China National Center for Bioinformation / Beijing Institute of Genomics, Chinese Academy of Sciences. The specific accession numbers are as follows: Metagenomics data: Genome Sequence Archive (GSA), accession number CRA028854, available at https://ngdc.cncb.ac.cn/gsa. Untargeted metabolomics data: OmiX, accession number OMIX011432, available at https://ngdc.cncb.ac.cn/omix. Single-cell sequencing data: OmiX, accession number OMIX011466, available at https://ngdc.cncb.ac.cn/omix.

## Abbreviations

CHEXs: Compact Single High-Energy X-Ray Source
CONV-RT: Conventional Dose Rate Radiotherapy
FLASH-RT: Flash Radiotherapy
PDD: Percent Depth Dose
NF-κB: Nuclear factor-kappa B
SCFAs: Short-chain fatty acids
