# Supplementary material for: Exploring the mechanisms of protective effect of high-energy X-ray FLASH radiotherapy on intestine through multi omics analysis
Source: Radiat Oncol. 2025 Nov 27;20:179. doi: 10.1186/s13014-025-02763-z (PMC12659162; doi:10.1186/s13014-025-02763-z)
Supplement: Supplementary file 1 — Supplementary Material 1 [file 13014_2025_2763_MOESM1_ESM.docx]

**Supplementary materials**

**Figure S1.**


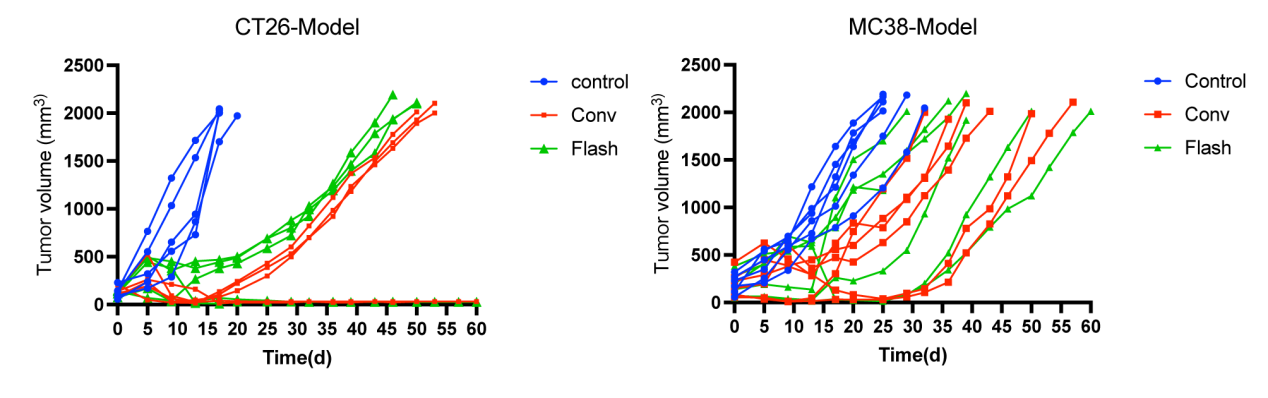


**Figure S1.**Tumor volume curves of individual mice in each group of the CT26/mc38 models.

**Figure S2**


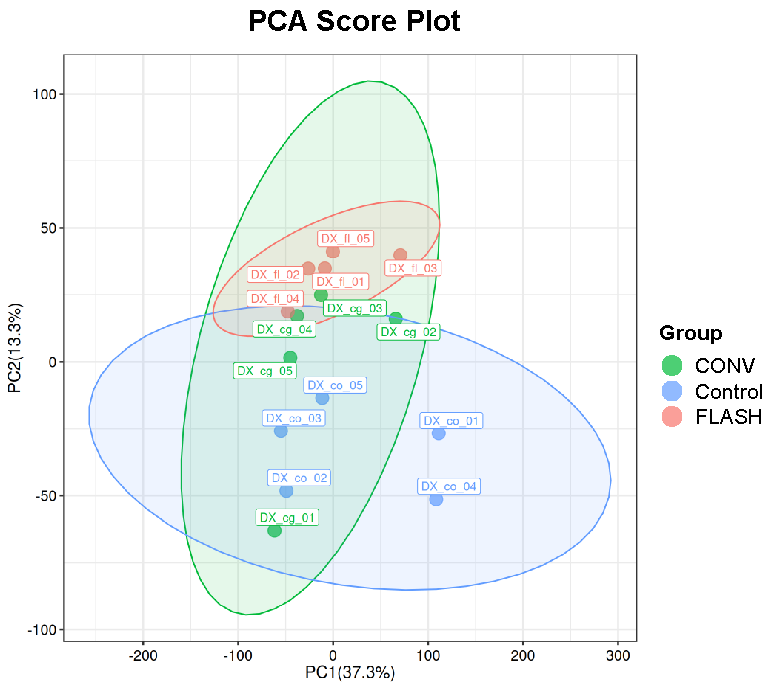


Figure S2. PCA score plots for each group.

**Figure S3**


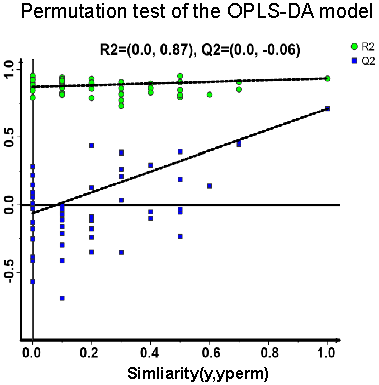


**Figure S3.** Permutation test of the OPLS-DA model for metabolomic profiling in positive ion mode. The x-axis quantifies the similarity between observed and permuted response variables (Similarity (y, yperm)), reflecting the degree of response variable randomization. The y-axis displays permutation-derived metrics: R² (green dots, model explanatory power) and Q² (blue dots, cross-validated predictive power). To evaluate overfitting, two criteria apply: Blue Q² dots in the upper-right region (high similarity) are higher than those on the left (low similarity); The regression line of Q² intersects the y-axis at a value < 0. For the positive ion mode dataset, both conditions are satisfied: upper-right Q² dots exceed left-side values, and the Q² regression intercept is < 0. These results confirm the OPLS-DA model is not overfitted and exhibits robust predictive performance.

**Figure S4**


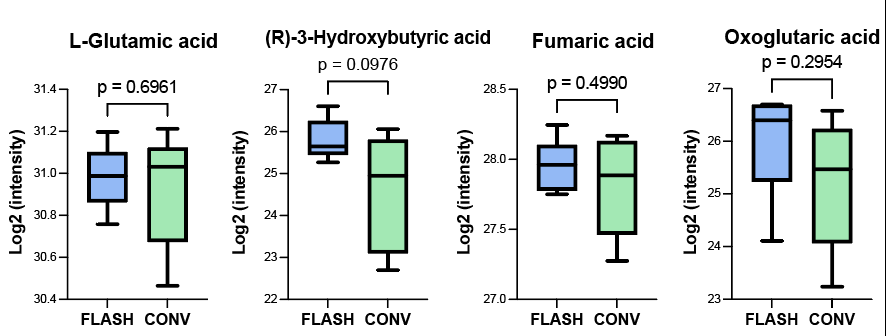


**Figure S4.** Bar chart of metabolite quantification in different groups. The differences between the two groups were analyzed using an unpaired t-test. The effect size is presented using Eta squared (η²).

**Figure S5**


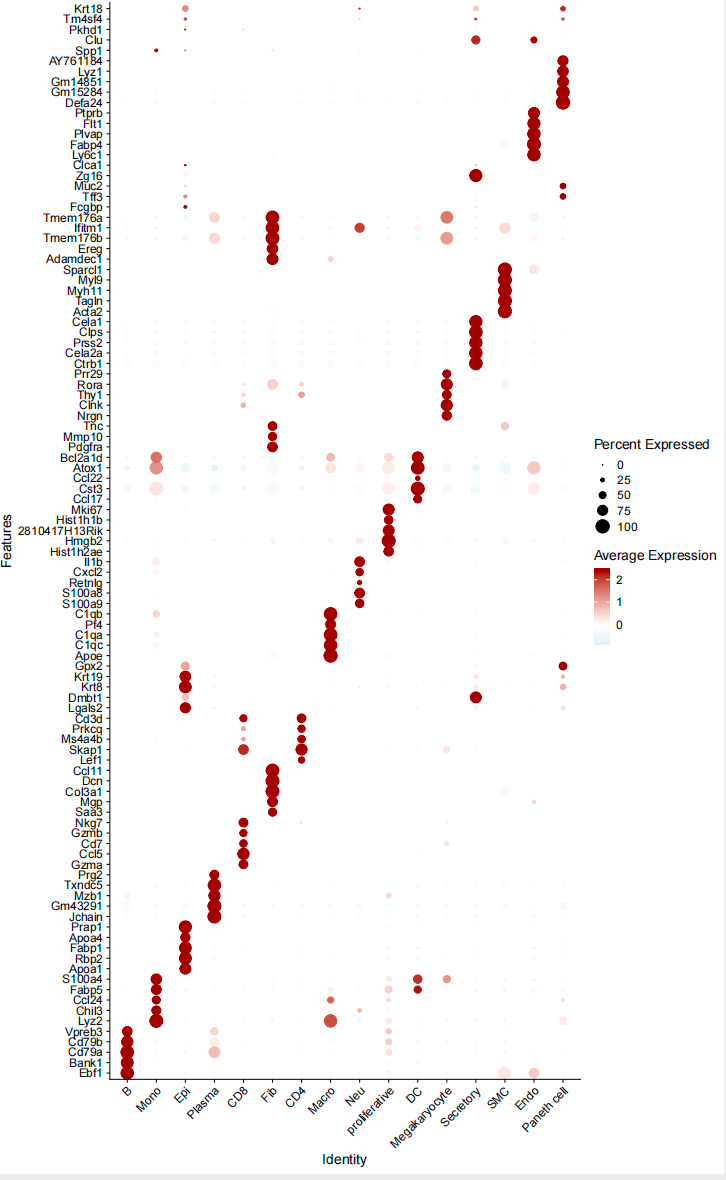


**Figure S5.** Expression profiles of marker genes for various cell types in intestinal tissues revealed by single - cell RNA sequencing (dot plot). The x - axis represents cell types, while the y - axis denotes candidate marker genes. The color gradient of the dots reflects the average expression level of the corresponding gene in each cell type (Average Expression; darker red indicates higher expression). The size of the dots indicates the percentage of cells expressing the gene (Percent Expressed; larger dots correspond to a higher proportion of expressing cells). This plot serves to identify cell - type - specific marker genes, providing a molecular basis for cell type annotation and subsequent functional analysis of single - cell sequencing data.

**Figure S6**


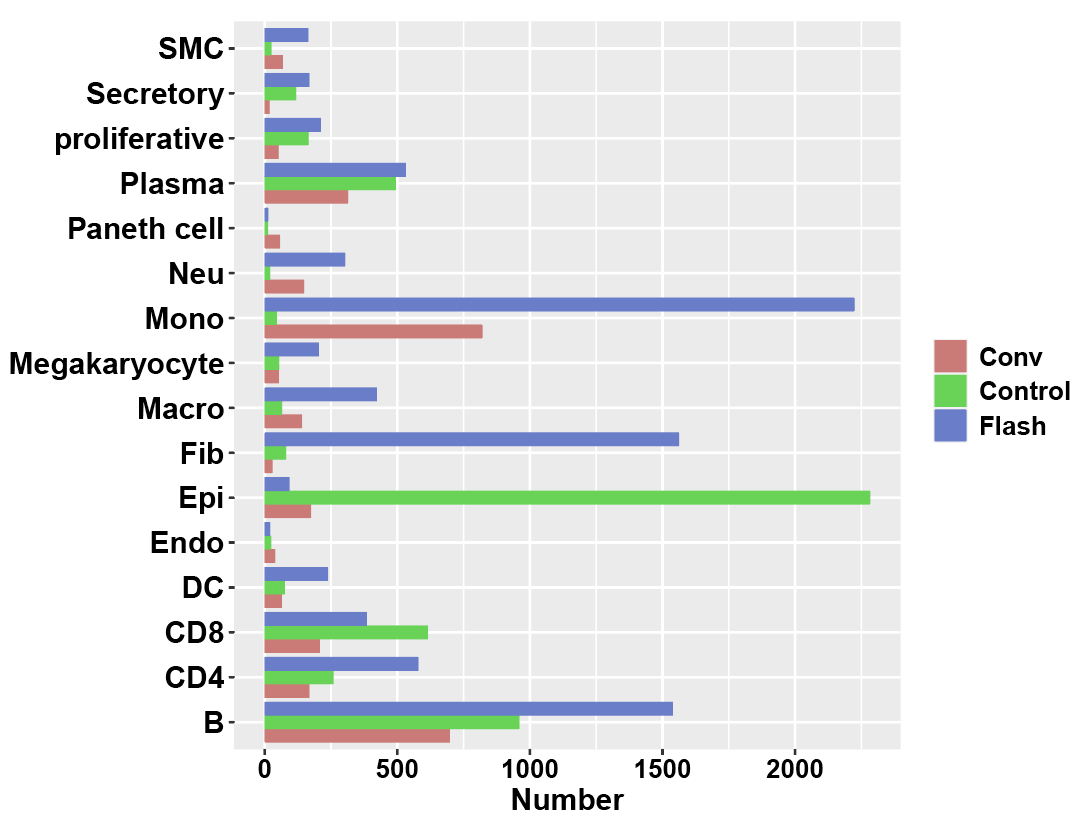


**Figure S6.** Cell number distribution across clusters in mouse intestinal single - cell RNA - seq, stratified by experimental group. The y - axis denotes cell cluster identities; the x - axis represents cell counts. Colors distinguish groups: Control (green), conventional radiotherapy (CONV, brown), and FLASH radiotherapy (FLASH, blue).
